# Integrated morphological, pomological, biochemical, and ISSR-based assessment of genetic diversity in wild sour orange (*Citrus aurantium* L.) genotypes from the Hassa district of Hatay, Türkiye

**DOI:** 10.1186/s40659-026-00708-8

**Published:** 2026-06-14

**Authors:** Yazgan Tunç

**Affiliations:** Republic of Türkiye Ministry of Agriculture and Forestry, General Directorate of Agricultural Research and Policies, Hatay Olive Research Institute Directorate, Hassa Station, 31700 Hassa, Hatay Türkiye

**Keywords:** *Citrus aurantium*, Wild sour orange, Genetic diversity, ISSR markers, Rootstock breeding, Hassa district (Hatay, Türkiye)

## Abstract

Sour orange (*Citrus aurantium* L.) is a hybrid species of considerable agronomic, medicinal, and industrial importance; however, its wild genetic resources remain largely underexplored. This study evaluated 24 naturally occurring wild sour orange genotypes collected from the Hassa district of Hatay, Türkiye, using an integrated framework combining morphological, pomological, biochemical, and ISSR molecular markers. The combined use of multi-trait phenotypic measurements and molecular markers enabled a comprehensive assessment of genetic and phenotypic diversity within this localized germplasm. Substantial variation was observed in fruit and leaf traits, indicating strong potential for the selection of contrasting ideotypes. Biochemical analyses revealed marked diversity in total phenolic and flavonoid contents, antioxidant capacity, and vitamin C levels, allowing the identification of genotypes that combine high nutritional quality with commercially acceptable fruit size. ISSR analysis produced a high polymorphism rate (75.72%), confirming pronounced genetic diversity within this micro-region. Multivariate analyses, including correlation, regression, principal component analysis, and hierarchical clustering visualized through heat maps, clearly discriminated genotypes according to fruit size, seed productivity, and phytochemical composition. Overall, the results highlight the presence of considerable genetic diversity within the Hassa district; however, given the geographically restricted sampling, these findings should be interpreted as representative of a localized germplasm rather than the entire species distribution. The study provides a practical basis for selecting superior genotypes for rootstock breeding, fresh fruit utilization, and nutraceutical applications.

**Supplementary Information:**

The online version contains supplementary material available at 10.1186/s40659-026-00708-8.

## Introduction

The common sour orange (*Citrus aurantium* L.) is a hybrid species resulting from a cross between mandarin (*C. reticulata* Blanco) and pummelo (*C. maxima* (Burm.) Merr), as reported by Wu et al. [62]. This hybrid origin has contributed to its considerable morphological and genetic variability, which underpins its broad adaptive capacity and diverse functional traits. Belonging to the Rutaceae family, sour orange is recognized as a key species within the genus Citrus [44]. Its phenotypic plasticity, combined with traits such as tolerance to diverse soil conditions, resistance to specific pathogens, and high compatibility with commercial citrus scions, has translated its genetic background into substantial agronomic value. Consequently, sour orange has become an essential component of citrus breeding and rootstock improvement programs, as well as a widely utilized species in citriculture systems worldwide [2, 29, 30].

Agronomically, sour orange remains one of the most widely used and reliable rootstocks in citrus cultivation due to its broad compatibility with scion cultivars and its tolerance to abiotic stresses such as drought and salinity, as well as its relative resistance to soil-borne pathogens including *Phytophthora* spp. and citrus nematodes [7, 9]. However, its susceptibility to Citrus Tristeza Virus (CTV) has historically limited its use in regions where the virus is prevalent [5, 10]. In Türkiye, including the Hatay region, CTV has been reported, although its distribution and impact vary depending on local ecological conditions and management practices. Consequently, the use of sour orange as a rootstock remains context-dependent, retaining its importance particularly in areas with low CTV pressure or in breeding programs. This duality highlights the continued relevance of sour orange both as a genetic resource for rootstock improvement and as a model for evaluating genotype performance under specific regional conditions. Beyond its rootstock role, sour orange is also utilized in the production of marmalades, essential oils, and flavoring agents, as well as for ornamental purposes due to its aromatic flowers and visually attractive fruits [34].

In addition to its agronomic importance, sour orange contains a range of bioactive compounds that contribute to its biochemical and nutritional value. The fruit is particularly rich in phenolic compounds and flavonoids (e.g., hesperidin and naringin), which are widely recognized for their antioxidant properties [46, 56]. These compounds are of particular interest in studies aiming to evaluate biochemical variation among genotypes. Therefore, assessing the diversity of such metabolites in sour orange germplasm may provide valuable insights for the identification of genotypes with enhanced phytochemical profiles and potential applications in breeding and functional food development.

Despite its economic and medicinal importance, the genetic diversity of sour orange remains underexplored, particularly in studies integrating morphological, pomological, biochemical, and molecular data. Previous research has demonstrated considerable phenotypic and genetic variability within Citrus aurantium and related taxa across different ecological regions. For instance, Kar et al. [36] reported significant altitude-driven variation in Citrus reticulata, highlighting the role of environmental gradients in shaping diversity. Similarly, Nguyen et al. [50] identified clear genetic structuring in citrus germplasm using SNP markers, while De Pasquale et al. [14] revealed substantial inter-accession variability in sour orange using ISSR and RAPD markers.

Morphological and pomological traits provide valuable insights into phenotypic diversity but are often influenced by environmental factors [45, 59]. In contrast, biochemical markers reflect metabolic variation and nutraceutical potential [40], while molecular markers enable direct assessment of genetic diversity at the DNA level. Among these, ISSR (Inter Simple Sequence Repeat) markers have been widely used due to their high reproducibility, multilocus nature, and ability to generate polymorphic banding patterns without requiring prior sequence information [22, 49]. Their effectiveness in diversity studies, particularly in wild or under-characterized germplasm, has been demonstrated in several studies [3, 6].

However, ISSR markers are dominant in nature and do not allow discrimination between homozygous and heterozygous loci, which may limit their resolution compared to codominant markers such as SSRs and SNPs. Despite this limitation, ISSRs remain a cost-effective and robust tool for preliminary genetic diversity assessment in wild germplasm, as in the present study.

In this context, the present study aims to elucidate the extent of genetic diversity among a set of sour orange genotypes by employing an integrative approach that combines morphological, pomological, biochemical, and molecular characterization. A total of 24 genotypes were evaluated using a diverse set of descriptors and ISSR primers to capture both phenotypic and genotypic variation.

To better understand the relationships among traits and genotypes, multivariate statistical approaches were applied.

The findings of this study are expected to provide insights into the existing variability within sour orange germplasm and may support future efforts in rootstock breeding, conservation, and the identification of genotypes with desirable agronomic and biochemical traits.

## Materials and methods

### Plant material

**Fig. 1:**
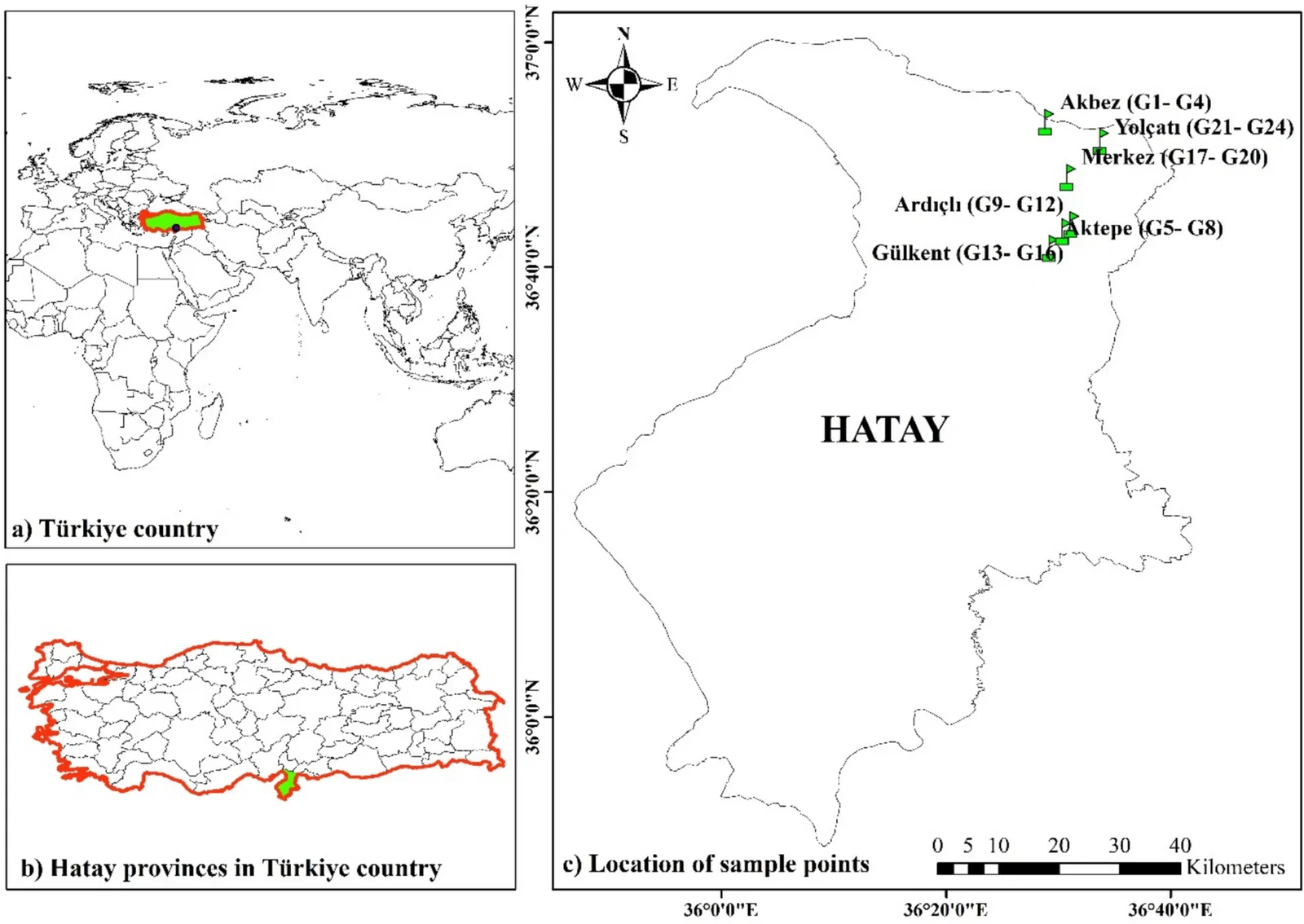
Geographic location of the sampling site for the studied sour orange genotypes. The map was produced using ArcGIS (Version 10.1) [17] by the author, Dr. Yazgan Tunç

Morphological, pomological, biochemical, and ISSR-based molecular variations of 24 naturally occurring wild sour orange genotypes were evaluated across six distinct locations (Akbez, Aktepe, Ardıçlı, Gülkent, Merkez, and Yolçatı) within the Hassa district of Hatay, Türkiye (Fig. 1). The sampling design was structured to capture the ecological and agro-environmental heterogeneity of the region, including variation in altitude, soil characteristics, precipitation regimes, and land-use history. Previous studies have demonstrated that even geographically restricted Citrus populations may exhibit considerable genetic and phenotypic variability due to evolutionary processes such as apomixis and limited gene flow [43, 60, 65]. Genotype selection was performed using a combined approach involving both field-based phenotypic observation and random sampling. Initially, trees exhibiting visible variation in growth habit, canopy structure, and fruit characteristics were identified during field surveys. Subsequently, individuals were selected randomly within each location while maintaining spatial separation criteria, ensuring the inclusion of representative variability and minimizing sampling bias. To minimize the likelihood of sampling clonally propagated or closely related individuals, a minimum distance of 200 m between sampled genotypes was maintained, consistent with spatial sampling criteria applied in similar studies on wild plant populations [37]. Although broader multi-regional sampling would provide a more comprehensive representation of the specie’s genetic diversity, the observed variation indicates that substantial intraspecific diversity is present within this micro-region. This highlights Hassa as a vital reservoir of sour orange genetic resources. Future studies incorporating wider geographic sampling and codominant molecular markers such as SSRs or SNPs will further refine the understanding of genetic structure in sour orange populations.

The geographical coordinates and altitude of the sampling sites were determined using a Global Positioning System (GPS) device (Garmin GPSMAP 67; Garmin Ltd., Olathe, Kansas, USA) and are presented in Table 1.

**Table 1:** Location and altitude information of the collection sites of sour orange genotypes investigated in the Hassa district of Hatay province, Türkiye

Location no	Neighborhood	Selected genotypes	Latitude (N)	Longitude (E)	Altitude (m a.s.l.)
1	Akbez	‘G1’- ‘G4’	36°52′54"	36°28′56"	401
2	Aktepe	‘G5’- ‘G8’	36°41′42"	36°29′18"	292
3	Ardıçlı	‘G9’- ‘G12’	36°43′48"	36°31′11"	309
4	Gülkent	‘G13’- ‘G16’	36°43′11"	36°30′28"	300
5	Merkez	‘G17’- ‘G20’	36°48′01"	36°30′52"	385
6	Yolçatı	‘G21’- ‘G24’	36°51′11"	36°33′48"	411

### The characteristics evaluated

Phenotypic diversity among the sour orange genotypes was assessed using 17 pomological, morphological, and biochemical traits (Table 2). Sampling was carried out separately in two consecutive growing seasons (2023 and 2024) at commercial harvest maturity. In each season, 50 leaves and 50 fruits were randomly collected from each genotype. To ensure sampling uniformity and minimize within-tree variation, samples were collected from the mid-canopy zone of each tree, representing the four cardinal directions (north, south, east, and west). Only healthy, undamaged, and fully developed organs were selected. The data obtained from both years were averaged prior to statistical analysis.

**Table 2:** Descriptive statistics for the pomological, morphological, and biochemical traits utilized in the studied sour orange genotypes

Traits	Abb	Unit	Min. (‘G.No.’)	Max. (‘G.No.’)	Mean	± SD	CV (%)
Pomological trait							
Fruit weight	V1	g	29.07 (‘G9’)	161.16 (‘G5’)	88.66	40.44	45.61
Fruit length	V2	mm	38.39 (‘G9’)	67.11 (‘G2’)	53.61	8.56	15.97
Fruit width	V3	mm	40.71 (‘G9’)	74.66 (‘G5’)	59.16	10.45	17.66
Epicarp thickness	V4	mm	1.15 (‘G10’)	2.94 (‘G2’)	1.84	0.45	24.46
Mesocarp thickness	V5	mm	1.38 (‘G10’)	8.15 (‘G24’)	4.24	1.72	40.57
Number of segments	V6	number	7 (‘G21’)	10 (‘G10’)	8.13	0.9	11.07
Number of seeds	V7	number	7 (‘G21’)	29 (‘G7’)	19.04	5.44	28.57
Morphological trait							
Leaf length	V8	mm	57.95 (‘G9’)	145.21 (‘G22’)	104.77	18.9	18.04
Leaf width	V9	mm	25.89 (‘G9’)	71.14 (‘G22’)	49.1	9.18	18.7
Leaf shape index	V10	ratio	1.51 (‘G2’)	2.68 (‘G8’)	2.18	0.24	11.01
Leaf area	V11	cm^2^	11.01 (‘G9’)	63.4 (‘G22’)	36.18	11.27	31.15
Petiole length	V12	mm	13.16 (‘G9’)	34.87 (‘G22’)	23.32	4.84	20.75
Petiole width	V13	mm	3.09 (‘G10’)	17.44 (‘G22’)	8.42	3.08	36.58
Biochemical trait							
Total phenolic content	V14	µg GAE/100 mL	103.74 (‘G18’)	200.78 (‘G11’)	145.08	30.58	21.08
Total antioxidant capacity	V15	%	24.5 (‘G18’)	47.6 (‘G5’)	36.02	7.7	21.38
Total flavonoid content	V16	µg CAE/100 mL	10.79 (‘G12’)	28.77 (‘G8’)	16.09	5.66	35.18
Vitamin C content	V17	g AAE/100 mL	16.41 (‘G20’)	37.26 (‘G21’)	26.26	6.28	23.91

*Max*: maximum; *Min*: minimum; *(‘G.No.’)*: genotype number; ± *SD*: standard deviation; *CV*: coefficient of variation

#### Morpho-pomological characteristics

Variables including fruit length, fruit width, epicarp thickness, mesocarp thickness, leaf length, leaf width, petiole length, and petiole width were measured using a digital caliper with ± 0.01 mm precision from the Insize brand (model no. 1108–300, Insize Co., ltd. Suzhou New District, 215,009, China). A precision scale branded Kern (model no. PCB 250–3, KERN & Sohn GmbH, Balingen, Germany) with ± 0.01 precision was used to measure fruit weight. The number of segments and the number of seeds were determined by counting. The leaf shape index was calculated by dividing the leaf length by the leaf width. Leaf area was detected using ImageJ® [32] software.

#### Biochemical characteristics

***Sample preparation*:** Biochemical analyses were conducted in three replicates, each consisting of 20 fruits. Before analysis, the fruits were cut in half and squashed using an electric citrus juicer (Sage 800CP). The obtained juice was then homogenized by vortexing at 1000 rpm for 3 min using a vortex mixer (DLAB MX-S). The homogenate was filtered through filter paper (Borox, 90 mm Ø), and the filtrate was transferred into Falcon tubes and stored at − 20 °C until biochemical analyses were performed [63].

***Total phenolic content:*** The total phenolic content was quantified using the Folin-Ciocalteu reagent. In brief, 500 μL of fresh fruit extract was mixed with 4.1 mL of distilled water, followed by the addition of 100 μL Folin-Ciocalteu’s reagent and 2% sodium carbonate (Na_2_CO_3_). The resulting mixture was incubated for 2 h in the dark at room temperature using an incubator (Elektromag M 3025 BP). Following incubation, the solution developed a bluish coloration and was measured spectrophotometrically at 760 nm (Shimadzu UV1800 spectrophotometer). Absorbance readings were converted into gallic acid equivalents (GAE) using a calibration curve constructed with gallic acid, and the final results were expressed as µg GAE/100 mL fresh weight (FW) [19]. The calibration curve exhibited a high degree of linearity (R^2^ ≥ 0.99), confirming the reliability and accuracy of the quantification.

***Total antioxidant capacity:*** The total antioxidant activity of sour orange genotypes was determined following the method described by Brand-Williams et al. [8]. For this purpose, a 0.26 mM DPPH (1,1-diphenyl-2-picryl-hydrazyl) solution was utilized. A volume of 100 μL of fruit extract was mixed with 2900 μL of ethyl alcohol and 1 mL of the DPPH solution. The mixture was then shaken at 250 rpm for 3 min at room temperature using a Biosan PSU-20i shaker, after which it was incubated in the dark at room temperature for 30 min. After incubation, the absorbance of the mixture was measured at 517 nm using a spectrophotometer, and the absorbance of the sample (CA_sample_) was recorded. A control solution prepared without fruit extract served as the blank, and its absorbance (CA_blank_) was also measured [47]. The antioxidant activity was calculated using the equation proposed by Garcia et al. [21] (Eq. 1).

1 $$ \% \,{\mathrm{inhibition}}\,{\mathrm{of}}\,{\mathrm{DPPH}} = [({\mathrm{CAblank}} - {\mathrm{CAsample}})/{\mathrm{CAblank}} \times 100 $$

***Total flavonoid content:*** The total flavonoid content in sour orange genotypes was assessed through a modified spectrophotometric method adapted from the procedure described by Chang et al. [11]. Briefly, 1000 μL of the fruit extract was combined with 3.3 mL of methanol (CH₃OH), followed by the sequential addition of 0.1 mL of 10% aluminum chloride hexahydrate (AlCl_3_·6H_2_O) and 0.1 mL of potassium acetate (CH_3_COOK). The resulting mixture was vortexed at 250 rpm for 3 min to achieve thorough homogenization. Absorbance was subsequently recorded at 415 nm using a spectrophotometer. Total flavonoid content was quantified using a standard calibration curve constructed with catechin, and the results were expressed as micrograms of catechin equivalents per 100 ml of fresh weight (µg CAE/100 mL FW). The calibration curve also showed strong linearity (R^2^ ≥ 0.99), ensuring the precision and reproducibility of the measurements.

***Vitamin C content:*** The vitamin C (ascorbic acid) content was quantified following the methodology described by Balta et al. [4]. Initially, fruit juice was diluted 100-fold with distilled water (1:100). A 1 mL aliquot of the diluted solution was then combined with five drops of TS-1 reagent and 10 mL of distilled water. A commercial test strip (Reflectoquant Total Sugar Test, Cat. No. 116136) was immersed in the reaction mixture for 2 s. Following a 10-min incubation period to allow for excess liquid removal, the strip was inserted into the reflectometer’s strip adapter (RQFlex Plus 10, Merck) for quantitative analysis. The obtained value was subsequently multiplied by the dilution factor, and results were expressed as grams of ascorbic acid equivalent per 100 ml of fresh weight (g AAE/100 mL FW).

### Molecular analysis through ISSR markers

Genomic DNA was extracted from young leaves of sour orange using the CTAB protocol as described by Doyle and Doyle [15]. DNA concentration and purity were evaluated spectrophotometrically (ONDA VIS-10 plus, ONDA Laboratori s.r.l.), and integrity was verified by electrophoresis on a 2% agarose gel alongside a 100 bp molecular weight marker (Thermo Fisher Scientific). The isolated DNA samples were stored at –20 °C until further use. For ISSR analysis, a larger set of primers was initially screened, and eight primers producing clear, reproducible, and polymorphic amplification patterns were selected for further analysis. This selection strategy ensured high-quality and reliable banding profiles. PCR reactions were carried out in a total volume of 15 µL, containing 2 µL of template DNA (20 ng), 1.5 µL of 10 × PCR buffer, 0.2 µL of Taq DNA polymerase (5 U/µL), 1 µL of dNTP mix (2.5 mM), 1.5 µL of MgCl_2_ (25 mM), 2 µL of ISSR primer (10 mM), and 6.8 µL of nuclease-free water. Amplifications were conducted following the thermal cycling protocol described by Uzun et al. [61], with minor optimization, comprising an initial denaturation at 94 °C for 2 min,38 cycles of denaturation at 94 °C for 1 min, annealing at 50 °C for 1 min, and extension at 72 °C for 1 min and 15 s; followed by a final extension step at 72 °C for 7 min. The annealing temperature of 50 °C was confirmed through preliminary trials to produce consistent and reproducible amplification across all primers and was therefore applied uniformly. The selected primers generated a total of 139 scorable bands, indicating a high level of polymorphism and providing sufficient resolution for reliable assessment of genetic diversity among the 24 genotypes.

### Statistical analysis

This research, conducted during the years 2023 and 2024, utilized the mean values derived from the two-year dataset. Multivariate statistical techniques were employed to assess genetic variation and similarities within the sour orange. To determine statistically significant differences among the 24 genotypes, one-way analysis of variance (ANOVA) was performed using the JMP® Pro 17 software [33]. Descriptive statistics including minimum, maximum, mean, and coefficient of variation (CV%) were also calculated using the same software. To explore relationships among the studied variables, multivariate analyses such as correlation matrix analysis, principal component analysis, and heat map analysis were conducted using Origin Pro® 2025b [52]. Pearson’s correlation coefficient was used to assess the strength and direction of associations between traits. Before constructing the heat map, all variables were normalized by converting them to Z-scores based on their respective means to eliminate scale differences. Heat map analysis was carried out using Ward’s method and Euclidean distance metric (Hammer et al. 2001). A two-dimensional principal component biplot was generated to visualize the dispersion of the genotypes. Furthermore, to examine the spatial distribution and regional patterns among genotypes collected from six distinct regions, additional 2D principal component analysis scatter plots were constructed. Moreover, multiple regression analysis was used to identify the explanatory variables that significantly influenced fruit and seed traits, which were treated as dependent variables. This analysis was conducted through the “stepwise” method under the “linear regression analysis” module in SPSS® software (SPSS Inc., Chicago, IL, USA) [16, 51],SPSS 2012).

For the ISSR analyses, banding patterns obtained via agarose gel electrophoresis and visualized using Kodak imaging systems (Eastman Kodak Company, Rochester, NY, USA) were scored as follows: band presence (1), absence (0), or no amplification (9). To ensure the reliability of the ISSR banding patterns, each primer–genotype combination was amplified in at least two independent PCR reactions performed on different days using the same thermal cycling conditions. The gels obtained from replicate amplifications were visually compared. Only clear, well-resolved bands that were consistently present in all replicate reactions were considered reproducible and were scored as “1” in the binary matrix. Faint, smeared, or ambiguous bands, as well as bands that appeared in only one of the replicate reactions, were regarded as non-reproducible and were excluded from the data set (scored as “0” or treated as missing data where appropriate). As a result of this procedure, the vast majority of scorable fragments consisted of highly reproducible bands, thereby enhancing the robustness and reliability of the ISSR dataset. The resulting binary data were processed using NTSYS version v.2.11X software [55]. Dendrograms depicting the genetic relationships among sour orange genotypes were created based on a similarity matrix using the Dice coefficient and UPGMA (Unweighted Pair Group Method with Arithmetic Mean) clustering method [57]. For each ISSR marker, the total number of bands, the number of polymorphic bands, and the polymorphism percentage were calculated. The polymorphism rate was determined using the formula outlined in Eq. 2 [66].

2 $$Polymorphism rate=\frac{Number of polymorphic bands}{Total number of bands}\times 100$$

### Analysis of Molecular Variance (AMOVA)

To further quantify the distribution of genetic variation, an Analysis of Molecular Variance (AMOVA) was performed using the binary ISSR data matrix (0/1) generated for all 24 genotypes (Supplementary Table S1). The six collection sites (Akbez, Aktepe, Ardıçlı, Gülkent, Merkez, and Yolçatı) were treated as separate populations. AMOVA partitioned the total molecular variance into “among-population” and “within-population” components, and the ΦPT (PhiPT) statistic—analogous to Wright’s FST—was calculated to estimate the degree of genetic differentiation among populations. Statistical significance was assessed using 999 permutations following the framework of Excoffier et al. [18].

## Results and discussion

### Descriptive statistics among genotypes

Descriptive statistics for the morphological characteristics assessed in the studied sour orange genotypes are summarized in Table 2. According to the one-way ANOVA results (*p* < 0.05), significant differences were detected among the genotypes, indicating considerable phenotypic variation. The highest variation was observed in fruit weight (45.61%), mesocarp thickness (40.57%), petiole width (36.58%), total flavonoid content (35.18%), and leaf area (31.15%), whereas the lowest variation was recorded in leaf length (18.04%), fruit width (17.66%), fruit length (15.97%), number of segments (11.07%), and leaf shape index (11.01%).

Notably, 11 out of the 17 evaluated traits (64.71%) exhibited coefficients of variation (CVs) exceeding 20.00%. While this threshold is commonly used as a general indicator of high variability [37], it should be interpreted with caution, as the magnitude of CV values may vary depending on the biological nature of the trait and its sensitivity to environmental influences. Traits with relatively higher CV values generally provide greater discriminatory power among genotypes, whereas lower CV values may reflect more stable and conserved characteristics.

Overall, the considerable variation observed among the sour orange genotypes indicates a high level of phenotypic diversity within the studied germplasm. This heterogeneity suggests that the evaluated genotypes may represent valuable genetic resources for future breeding and selection programs.

In terms of fruit-related traits (Table 3), fruit weight ranged from 29.07 g in genotype ‘G9’ to 161.16 g in ‘G5’, indicating more than a fivefold difference among genotypes. Fruit length varied between 38.39 mm (‘G9’) and 67.11 mm (‘G2’), while fruit width ranged from 40.71 mm (‘G9’) to 74.66 mm (‘G5’). These results demonstrate substantial morphological divergence in fruit size and shape within the studied germplasm. Epicarp thickness ranged from 1.15 mm to 2.94 mm, and mesocarp thickness varied between 1.38 mm and 8.15 mm, indicating considerable diversity in rind structure and pulp development, which may directly influence fruit texture and juice yield. Seed number also showed marked variation (7–29), which is particularly relevant for rootstock potential, as higher seed production is a desirable trait for propagation efficiency. The observed ranges are generally consistent with previous studies conducted in different citrus-growing regions. For instance, Gogorcena and Ortiz [25] reported fruit weight ranging from 45.9 to 174.8 g and seed number between 7 and 28 in Spain, while Dahmani et al. [13] documented comparable ranges for fruit size and rind characteristics in Morocco. Similarly, Rohini et al. [54] reported broader variation in fruit traits in Indian germplasm. However, differences in the magnitude of variation among studies may be attributed to genetic diversity, environmental conditions, and methodological differences. The relatively wide variation observed in the present study suggests that the sampled genotypes harbor substantial phenotypic diversity, likely influenced by both local ecological conditions and underlying genetic factors. Overall, the diversity in fruit-related traits highlights the potential of these genotypes for use in breeding programs targeting fruit quality, rootstock development, and adaptation to specific agroecological conditions.

**Table 3:** Pomological characteristics of sour orange genotypes

Genotypes	Fruit weight (g)	Fruit length (mm)	Fruit width (mm)	Epicarp thickness (mm)	Mesocarp thickness (mm)	Number of segments (number)	Number of seeds (number)
‘G1’	61.76 ± 2.7^hi^	47.77 ± 085^ g−i^	53.97 ± 1.05^f−h^	1.87 ± 0.07^d−h^	4.06 ± 0.36^c−h^	8.30 ± 0.33^b−e^	15.30 ± 1.94^d−f^
‘G2’	133.49 ± 7.90^a−c^	67.10 ± 1.67^a^	72.07 ± 2.01^ab^	2.94 ± 0.17^a^	5.48 ± 0.48^b−d^	7.90 ± 0.35^b−e^	17.50 ± 2.16^b−f^
‘G3’	120.07 ± 6.68^ cd^	56.92 ± 1.52^d−f^	66.89 ± 1.93^a−d^	1.87 ± 0.13^c−h^	2.91 ± 0.24^d−i^	8.60 ± 0.27^b−d^	16.40 ± 1.93^c−f^
‘G4’	33.78 ± 3.12^jk^	41.01 ± 1.63^ij^	43.16 ± 1.85^jk^	1.34 ± 0.14^ h−j^	4.87 ± 0.46^b−e^	7.30 ± 0.42^c−e^	17.50 ± 1.95^b−f^
‘G5’	161.16 ± 5.89^a^	65.23 ± 1.20^ab^	74.66 ± 1.05^a^	2.08 ± 0.09^b−g^	3.91 ± 0.35^c−i^	7.90 ± 0.28^b−e^	20.80 ± 2.56^a−e^
‘G6’	55.82 ± 5.35^i−k^	46.97 ± 1.42^ g−i^	50.50 ± 1.77^ h−j^	1.37 ± 0.05^ h−j^	4.77 ± 0.34^b−e^	8.90 ± 0.23^a−c^	27.60 ± 2.21^ab^
‘G7’	136.49 ± 6.13^a−c^	64.17 ± 0.71^a−c^	69.28 ± 1.57^a−c^	2.21 ± 0.13^b−f^	4.12 ± 0.32^c−h^	8.70 ± 0.30^b−d^	29.20 ± 1.94^a^
‘G8’	91.15 ± 5.59^e−g^	57.22 ± 1.19^c−f^	60.53 ± 1.71^d−f^	1.60 ± 0.06^f−j^	4.72 ± 0.53^b−f^	7.40 ± 0.27^b−e^	20.40 ± 2.33^a−e^
‘G9’	29.07 ± 2.57^ k^	38.39 ± 1.09^j^	40.70 ± 1.32^ k^	1.35 ± 0.10^ h−j^	5.94 ± 0.41^a−c^	7.20 ± 0.25^d−e^	12.80 ± 2.91^ef^
‘G10’	105.42 ± 4.07^d−f^	56.46 ± 1.41^d−f^	60.39 ± 0.80^d−f^	1.15 ± 0.11^j^	1.37 ± 0.11^i^	10.40 ± 0.22^a^	10.70 ± 0.91^ef^
‘G11’	52.16 ± 3.70^i−k^	46.85 ± 1.27^ g−i^	51.80 ± 1.50^ g−i^	1.21 ± 0.07^ij^	7.03 ± 0.74^ab^	8.10 ± 0.18^b−e^	19.50 ± 2.53^a−e^
‘G12’	87.73 ± 6.10^f−h^	56.66 ± 1.94^d−f^	62.35 ± 1.88^c−e^	2.60 ± 0.17^ab^	4.67 ± 0.96^b−f^	7.80 ± 0.25^b−e^	14.70 ± 1.45^d−f^
‘G13’	58.89 ± 4.16^ij^	45.17 ± 1.21^ h−j^	50.33 ± 1.49^ h−j^	1.43 ± 0.10^ h−j^	2.40 ± 0.12^e−i^	8.20 ± 0.42^b−e^	14.70 ± 2.09^d−f^
‘G14’	54.51 ± 4.95^i−k^	45.92 ± 1.55^hi^	48.51 ± 1.74^ h−k^	1.64 ± 0.12^f−j^	1.65 ± 0.20^hi^	7.90 ± 0.23^b−e^	15.60 ± 1.70^d−f^
‘G15’	75.55 ± 4.64^ g−i^	51.11 ± 1.10^f−h^	59.26 ± 1.56^d−g^	1.83 ± 0.17^d−h^	4.97 ± 0.40^b−e^	8.50 ± 0.27^b−d^	26.50 ± 1.65^a−c^
‘G16’	133.30 ± 9.06^bc^	64.78 ± 2.11^ab^	73.31 ± 2.33^ab^	2.26 ± 0.13^b−e^	5.61 ± 0.65^a−c^	9.00 ± 0.47^ab^	27.70 ± 3.03^ab^
‘G17’	93.68 ± 2.33^d−g^	53.72 ± 0.69^e−g^	59.65 ± 0.67^d−g^	1.66 ± 0.09^e−j^	2.83 ± 0.26^e−i^	8.50 ± 0.22^b−d^	20.40 ± 1.07^a−e^
‘G18’	62.78 ± 2.57^hi^	51.34 ± 0.93^f−h^	54.42 ± 0.80^e−h^	1.88 ± 0.09^c−h^	4.78 ± 0.29^b−e^	7.20 ± 0.25^de^	20.40 ± 2.03^a−e^
‘G19’	116.20 ± 6.38^c−e^	59.47 ± 1.21^b−e^	65.01 ± 1.26^b−d^	2.14 ± 0.13^b−f^	4.56 ± 0.62^b−g^	8.20 ± 0.36^b−e^	29.70 ± 2.70^a^
‘G20’	137.32 ± 7.30^a−c^	61.61 ± 1.54^a−d^	67.51 ± 1.33^a−d^	1.83 ± 0.10^d−i^	2.16 ± 0.16^f−i^	8.80 ± 0.29^a−d^	24.00 ± 1.80^a−d^
‘G21’	33.51 ± 2.04^jk^	41.08 ± 0.86^ij^	44.77 ± 1.25^i−k^	1.52 ± 0.08^ g−j^	5.74 ± 0.78^a−c^	6.70 ± 0.30^e^	7.10 ± 1.02^f^
‘G22’	72.82 ± 4.64^ g−i^	49.25 ± 1.02^gh^	53.56 ± 1.43^f−h^	1.80 ± 0.11^d−i^	2.07 ± 0.27^ g−i^	7.40 ± 0.37^b−e^	17.70 ± 1.77^b−f^
‘G23’	160.99 ± 4.47^ab^	64.80 ± 0.77^ab^	74.49 ± 1.55^a^	2.48 ± 0.15^a−c^	2.59 ± 0.22^e−i^	9.00 ± 0.37^ab^	21.20 ± 1.82^a−e^
‘G24’	113.41 ± 7.20^c−f^	61.56 ± 2.40^a−d^	73.12 ± 2.79^ab^	2.30 ± 0.16^b−d^	8.14 ± 1.04^a^	8.00 ± 0.33^b−e^	16.70 ± 2.06^c−f^

Levels not connected by the same letters are significantly different (*p* < *0.05*)

Morphological traits also exhibited substantial diversity (Table 4). Leaf length varied from 57.95 mm in ‘G9’ to 145.21 mm in ‘G22’, and leaf width ranged from 25.89 mm (‘G9’) to 71.14 mm (‘G22’), indicating that ‘G22’ consistently displayed the largest leaf dimensions. The leaf shape index ranged from 1.51 (‘G2’) to 2.68 (‘G8’), reflecting clear genotypic differences in leaf morphology. Leaf area, an important indicator of photosynthetic capacity, ranged from 11.01 cm^2^ to 63.4 cm^2^, further supporting the superior vegetative vigor of ‘G22’. Petiole length and width also showed considerable variation, which may influence leaf mechanical support and flexibility. When compared with previous studies, the observed ranges are generally consistent with those reported in citrus germplasm. For instance, Gogorcena and Ortiz [25] documented comparable variation in leaf dimensions in Spain, while Rohini et al. [54] reported similar variability in Indian genotypes. However, the slightly broader variation observed in the present study may reflect the heterogeneous ecological conditions of the Hassa region, as well as the inclusion of wild genotypes with diverse genetic backgrounds. Overall, the substantial variation in leaf morphology highlights the presence of significant phenotypic diversity, which may have important implications for photosynthetic efficiency, environmental adaptation, and genotype selection in breeding programs.

**Table 4:** Leaf morphology of sour orange genotypes

	Leaf length (mm)	Leaf width (mm)	Leaf shape index	Leaf area (cm^2^)	Petiole length (mm)	Petiole width (mm)
‘G1’	92.18 ± 6.81^ef^	42.70 ± 3.17^d−g^	2.17 ± 0.06^a−e^	27.53 ± 3.64^d−f^	21.96 ± 2.06^b−e^	6.57 ± 0.99^bc^
‘G2’	92.93 ± 7.12^ef^	61.83 ± 4.88^ab^	1.50 ± 0.03^f^	41.92 ± 6.23^b−e^	25.83 ± 1.78^a−d^	13.85 ± 3.14^ab^
‘G3’	99.28 ± 4.56^d−f^	47.46 ± 2.75^b−f^	2.12 ± 0.10^b−e^	32.80 ± 2.78^c−e^	21.24 ± 2.37^b−e^	9.62 ± 2.90^a−c^
‘G4’	131.68 ± 6.50^a−c^	57.87 ± 3.28^a−d^	2.28 ± 0.05^a−d^	51.21 ± 4.84^a−c^	29.38 ± 2.63^a−c^	10.79 ± 1.76^a−c^
‘G5’	108.44 ± 7.05^b−f^	51.98 ± 2.88^b−f^	2.08 ± 0.07^b−e^	39.81 ± 4.41^b−e^	25.55 ± 1.81^b−d^	10.35 ± 0.81^a−c^
‘G6’	105.45 ± 3.48^b−f^	49.76 ± 2.22^b−f^	2.13 ± 0.04^b−e^	35.02 ± 2.35^d−e^	21.74 ± 1.02^b−e^	7.31 ± 0.86^bc^
‘G7’	102.61 ± 8.01^c−f^	50.42 ± 3.75^b−f^	2.06 ± 0.11^b−e^	35.60 ± 4.97^b−e^	24.74 ± 1.74^b−d^	8.73 ± 2.03^bc^
‘G8’	129.18 ± 5.49^a−d^	49.06 ± 2.83^b−f^	2.68 ± 0.13^a^	42.41 ± 3.66^a−e^	27.03 ± 1.49^a−d^	6.02 ± 0.85^bc^
‘G9’	57.94 ± 5.00^ g^	25.89 ± 1.91^ g^	2.29 ± 0.17^a−d^	11.01 ± 1.42^f^	13.16 ± 1.50^e^	4.14 ± 0.48^c^
‘G10’	92.09 ± 1.87^ef^	41.55 ± 1.92^d−g^	2.25 ± 0.10^a−e^	25.66 ± 1.64^d−f^	13.27 ± 0.91^e^	3.09 ± 0.18^c^
‘G11’	90.85 ± 4.38^ef^	47.42 ± 1.64^b−f^	1.91 ± 0.06^d−f^	33.64 ± 5.22^c−e^	22.49 ± 1.59^b−d^	9.45 ± 1.41^a−c^
‘G12’	94.72 ± 5.91^ef^	37.99 ± 1.77^ fg^	2.49 ± 0.11^ab^	24.15 ± 2.35^d−f^	22.19 ± 1.94^b−e^	6.35 ± 0.80^bc^
‘G13’	103.00 ± 4.57^c−f^	42.41 ± 1.76^d−g^	2.44 ± 0.09^a−c^	29.78 ± 2.16^d−f^	23.22 ± 1.57^b−d^	5.74 ± 0.36^bc^
‘G14’	108.96 ± 6.90^b−f^	49.10 ± 3.72^b−f^	2.24 ± 0.06^a−e^	36.82 ± 4.57^b−e^	26.14 ± 2.21^a−d^	9.15 ± 2.00^bc^
‘G15’	100.66 ± 6.02^c−f^	56.71 ± 2.27^a−e^	1.76 ± 0.06^ef^	40.56 ± 3.71^b−e^	21.04 ± 1.98^c−e^	8.18 ± 1.20^bc^
‘G16’	104.07 ± 6.29^c−f^	44.30 ± 2.49^c−f^	2.37 ± 0.11^a−d^	31.19 ± 3.37^c−f^	21.21 ± 1.30^c−e^	5.19 ± 0.49^c^
‘G17’	90.97 ± 6.05^ef^	40.78 ± 4.48^e−g^	2.36 ± 0.16^a−d^	26.21 ± 4.37^d−f^	20.25 ± 1.67^de^	6.99 ± 0.84^bc^
‘G18’	108.74 ± 6.19^b−f^	50.36 ± 3.02^b−f^	2.18 ± 0.10^a−e^	37.72 ± 3.80^b−e^	21.12 ± 2.07^c−e^	9.11 ± 1.49^bc^
‘G19’	116.08 ± 7.09^a−e^	49.84 ± 2.85^b−f^	2.32 ± 0.05^a−d^	40.06 ± 4.41^b−e^	25.15 ± 1.32^b−d^	7.94 ± 0.61^bc^
‘G20’	137.14 ± 9.20^ab^	60.00 ± 3.47^a−c^	2.28 ± 0.06^a−e^	56.47 ± 6.97^ab^	28.81 ± 2.06^a−d^	7.48 ± 1.03^bc^
‘G21’	118.95 ± 4.04^a−e^	52.34 ± 1.42^b−f^	2.27 ± 0.05^a−e^	41.82 ± 2.55^b−e^	30.27 ± 1.09^ab^	9.63 ± 0.57^a−c^
‘G22’	145.20 ± 7.36^a^	71.14 ± 7.87^a^	2.17 ± 0.16^a−e^	63.40 ± 5.30^a^	34.87 ± 1.18^a^	17.44 ± 3.72^a^
‘G23’	78.25 ± 4.28^ fg^	41.11 ± 2.86^d−g^	1.98 ± 0.15^c−f^	22.13 ± 1.88^ef^	19.96 ± 1.19^de^	6.63 ± 0.91^bc^
‘G24’	112.17 ± 6.78^b−e^	55.85 ± 2.40^a−e^	2.00 ± 0.07^b−f^	43.66 ± 4.22^a−d^	22.90 ± 1.87^b−d^	11.04 ± 1.47^a−c^

Levels not connected by the same letters are significantly different (*p* < *0.05*)

Biochemical characteristics demonstrated equally pronounced variation (Table 5). Total phenolic content ranged from 103.74 to 200.78 µg GAE/100 mL, while total flavonoid content varied between 10.79 and 28.77 µg CAE/100 mL, indicating considerable diversity in secondary metabolite accumulation among genotypes. Total antioxidant capacity also exhibited notable variation (24.5–47.6%), suggesting differences in oxidative stress mitigation potential. Vitamin C content ranged from 16.41 to 37.26 g AAE/100 mL, showing more than a twofold difference and highlighting specific genotypes such as ‘G21’ as promising candidates for nutritional enhancement. The observed variation in biochemical traits can be attributed to both genetic and environmental factors. Phenolic and flavonoid compounds are known to be highly responsive to environmental conditions such as light intensity, temperature, and water availability, as well as to genotype-specific metabolic regulation. The relatively high phenolic content observed in certain genotypes (e.g., ‘G11’) may reflect enhanced activation of the phenylpropanoid pathway, while variation in antioxidant capacity likely results from the combined effects of multiple bioactive compounds. When compared with previous studies, the values obtained in this study are generally within the range reported for citrus species; however, differences in magnitude may arise from variations in genotype, ecological conditions, and analytical methodologies. For example, Ghafar et al. [23] reported higher phenolic contents in certain Citrus species, which may be related to species-specific metabolic capacity, while KhlongKhlaeo et al. [38] highlighted considerable variability in antioxidant activity depending on extraction and assay conditions. Overall, the substantial biochemical diversity observed among the studied genotypes highlights their potential for use in breeding programs targeting nutraceutical quality, as well as for the development of functional food products. The identification of genotypes combining high phenolic, flavonoid, and vitamin C contents provides a valuable basis for future selection and utilization strategies.

**Table 5:** Biochemical contents of sour orange genotypes

Genotypes	Total phenolic content (µg GAE/100 mL)	Total antioxidant capacity (% inhibition)	Total flavonoid content (µg CAE/100 mL)	Vitamin C content (g AAE/100 mL)
‘G1’	164.99 ± 5.48^e^	27.70 ± 3.34^ k−n^	13.11 ± 1.30^f−h^	29.16 ± 2.36^d−f^
‘G2’	108.17 ± 4.03^ l^	45.30 ± 3.76^a−c^	13.92 ± 2.03^e−h^	18.73 ± 5.46^ lm^
‘G3’	104.42 ± 8.84^ m^	29.00 ± 1.97^j−m^	10.89 ± 1.31^ h^	17.57 ± 2.20^ m^
‘G4’	172.15 ± 8.96^d^	31.30 ± 3.46^ij^	22.71 ± 1.87^b^	29.93 ± 2.99^c−f^
‘G5’	157.49 ± 4.92^f^	47.60 ± 1.05^a^	28.67 ± 3..33^a^	32.24 ± 5.19^ cd^
‘G6’	179.53 ± 5.66^c^	31.00 ± 2.46^ij^	20.69 ± 2.17^bc^	25.29 ± 4.62^ h−j^
‘G7’	112.37 ± 6.28^ k^	42.60 ± 3.73^c−e^	11.30 ± 0.36^gh^	28.00 ± 2.81^f−h^
‘G8’	182.71 ± 6.31^b^	25.20 ± 1.88^no^	28.77 ± 3.98^a^	24.52 ± 4.27^i−k^
‘G9’	162.15 ± 9.68^e^	32.60 ± 4.85^hi^	14.33 ± e-2.74^ g^	22.59 ± 4.63^jk^
‘G10’	118.74 ± 6.23^j^	45.70 ± 2.72^a−c^	13.01 ± 2.28f-^h^	26.84 ± 3.77^f−i^
‘G11’	200.78 ± 4.97^a^	34.50 ± 2.75^gh^	20.08 ± 3.24^bc^	26.84 ± 5.04^f−i^
‘G12’	132.15 ± 8.34^ h^	42.00 ± 3.35^de^	10.79 ± 1.17^ h^	35.72 ± 1.76^ab^
‘G13’	125.67 ± 6.54^i^	29.50 ± 2.66^i−l^	18.77 ± 0.80^ cd^	17.19 ± 1.39^ m^
‘G14’	143.96 ± 5.53^ g^	42.70 ± 2.81^c−e^	12.61 ± 1.36^f−h^	24.14 ± 4.91^i−k^
‘G15’	146.01 ± 9.50^ g^	26.70 ± 1.83^ l−o^	13.52 ± 1.79^e−h^	28.77 ± 5.94^e−g^
‘G16’	110.44 ± 9.99^kl^	39.60 ± 1.30^ef^	13.52 ± 1.88^e−h^	24.14 ± 2.45^i−k^
‘G17’	163.74 ± 7.93^e^	44.30 ± 3.99^b−d^	10.99 ± 2.01^ h^	25.68 ± 2.77^ g−j^
‘G18’	103.74 ± 9.89^ m^	24.50 ± 2.31^o^	14.73 ± 2.36^ef^	36.49 ± 5.33^a^
‘G19’	164.87 ± 7.80^e^	26.30 ± 4.12^ mn^	11.90 ± 2.70^f−h^	31.86 ± 3.24^c−e^
‘G20’	135.21 ± 7.62^ h^	46.40 ± 3.00^ab^	16.45 ± 1.22^de^	16.41 ± 4.13^ m^
‘G21’	182.03 ± 7.40^bc^	42.10 ± 1.41^de^	26.95 ± 4.80^a^	37.26 ± 3.40^a^
‘G22’	184.87 ± 8.51^b^	30.70 ± 4.04^i−k^	14.73 ± 0.40^ef^	32.63 ± 4.13^bc^
‘G23’	104.76 ± 7.86^ m^	40.00 ± 4.52^ef^	11.60 ± 0.99^f−h^	16.80 ± 3.45^ m^
‘G24’	120.90 ± 6.80^j^	37.10 ± 2.28^ fg^	12.21 ± 3.08^f−h^	21.43 ± 4.90^kl^

Levels not connected by the same letters are significantly different (*p* < *0.05*)

Taken together, the results demonstrate that the studied sour orange genotypes differ markedly in fruit size, vegetative vigor, and biochemical composition. Genotype ‘G9’ was repeatedly associated with minimum values in fruit weight, fruit length, fruit width, leaf dimensions, and leaf area, suggesting it may represent a dwarf or less vigorous genotype. Conversely, ‘G22’ displayed maximum values in all recorded morphological traits, indicating high vegetative growth potential. ‘G5’ distinguished itself by having the heaviest fruits and the highest antioxidant capacity, while ‘G21’ was notable for high vitamin C content and low seed number. Genotype ‘G11’ stood out for its high total phenolic content, and ‘G8’ for its high flavonoid concentration. The diverse trait expression among genotypes underscores their potential for use in breeding programs targeting specific agronomic or nutritional traits. Strategic hybridization among genotypes with complementary attributes, such as combining the high fruit quality and antioxidant activity of ‘G5’ with the vegetative vigor of ‘G22’ and the nutritional richness of ‘G21’, could enhance cultivar development efforts.

The findings obtained in this study generally align with the results of similar research conducted in different countries. However, slight variations observed in some pomological, morphological, and biochemical characteristics are likely attributable to several factors, including differences in genetic material (i.e., the diversity of genotypes or species used in the studies), variations in methodological approaches (such as sampling time, analytical techniques, and precision of measurement tools), and the distinct ecological conditions under which the studies were conducted (e.g., climate, soil structure, and other environmental factors). These results suggest that the examined traits may be influenced by both environmental conditions and genetic variation and that comparative studies of this nature can contribute to a better understanding of intra-species variability. Furthermore, this diversity holds significant potential for the conservation of genetic resources and for identifying superior genotypes that may be utilized in future breeding programs.

The high levels of phenotypic diversity identified in this study, particularly in fruit morphology, leaf characteristics, and internal biochemical attributes, are consistent with the findings of Ferrer et al. [20], who suggested that morphological and aromatic variation in sour orange may be partly driven by mutation-based diversification. However, the observed variability cannot be attributed solely to somaclonal variation,rather, it is likely influenced by multiple factors, including genetic differentiation, localized gene flow, and environmental conditions. Therefore, while mutation-driven diversification may contribute to phenotypic variation, the underlying mechanisms are likely to be more complex.

**Fig. 2:**
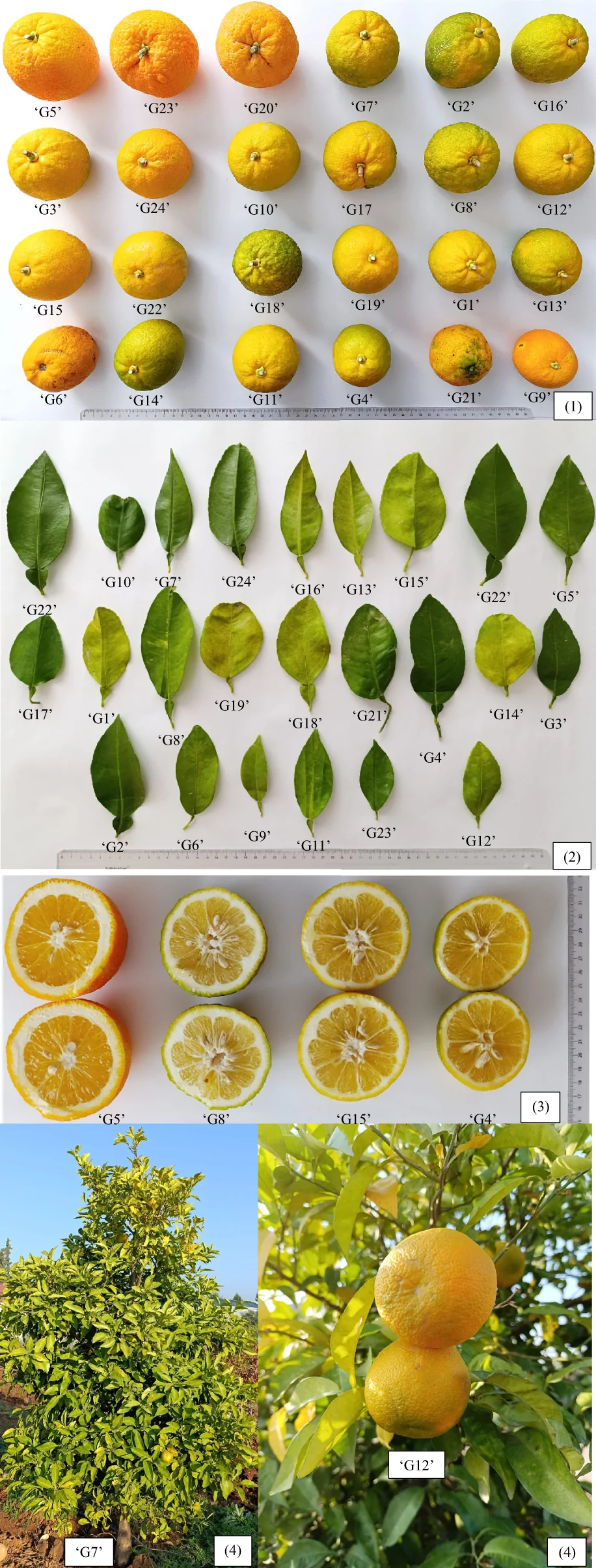
Morphological diversity among sour orange genotypes, including variation in fruit characteristics (size, shape, rind traits) (**1**), leaf morphology (size, shape, colour) (**2**), internal fruit structure (pulp and seed traits) (**3**), and tree-level canopy architecture (**4**). Scale bars are provided for reference. This photograph was taken as part of the present study and is provided by the author, Dr. Yazgan Tunç; it is free from any third-party copyright restrictions

The considerable phenotypic variability in fruit, leaf, internal fruit structure, and canopy traits among the studied sour orange genotypes is clearly demonstrated in Fig. 2.

### Correlation matrix analysis (CMA)

**Fig. 3:**
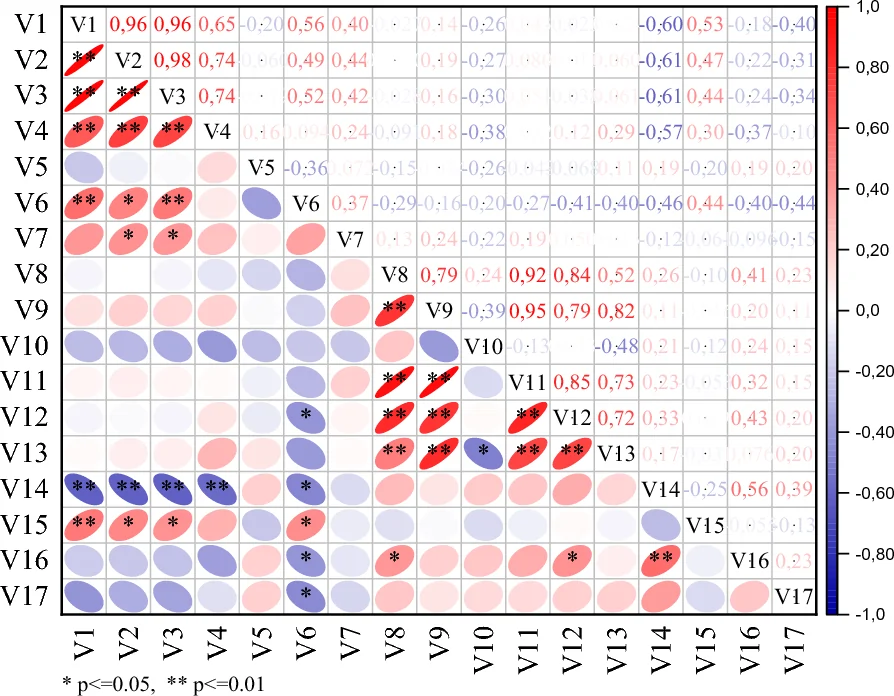
Simple correlations between the pomological, morphological, and biochemical variables utilized in the studied sour orange genotypes. For abbreviations, please see Table 2

The correlation analysis results revealed statistically significant relationships among several pomological and biochemical traits (Fig. 3 and Supplementary Table S2). Fruit weight exhibited strong positive correlations with fruit length (*r* = 0.96**), fruit width (*r* = 0.96**), epicarp thickness (*r* = 0.65**), number of segments (*r* = 0.56**), and total antioxidant capacity (*r* = 0.53**), indicating that fruit size is closely associated with both dimensional characteristics and specific functional properties. Notably, the positive relationship with antioxidant capacity suggests that larger fruits may possess higher levels of antioxidant activity, which is particularly relevant from a human health perspective.

However, fruit weight was also negatively correlated with total phenolic content (*r* = –0.60**), and a similar negative correlation was observed between fruit length and total phenolic content (*r* = –0.61**). These findings imply that phenolic compounds tend to be more concentrated in small- to medium-sized fruits and become diluted as fruit size increases. This may point to a physiological balance between fruit development and phenolic biosynthesis. Therefore, if the objective is the production of functional foods or pharmaceutical applications, minor fruit genotypes may be preferred due to their potentially richer phytochemical content.

Fruit length showed significant positive correlations with fruit width (*r* = 0.98**), epicarp thickness (*r* = 0.74**), number of segments (*r* = 0.49*), number of seeds (*r* = 0.44*), and total antioxidant capacity (*r* = 0.47*). These findings collectively indicate that as fruits become lengthier (and typically larger), their biochemical profiles shift toward higher antioxidant capacity. This trade-off should be considered in breeding programs depending on the target end-use. The relationship with seed number is particularly important for breeding programs and rootstock selection, where seed production capacity may be a desirable trait.

Total phenolic content showed a strong negative correlation with fruit width (*r* = –0.61**) and a positive correlation with total flavonoid content (*r* = 0.56**). This suggests a potential inverse relationship between fruit size and phenolic concentration, and a synergistic association between phenolics and flavonoids, possibly due to shared or co-regulated biosynthetic pathways. These compounds are of significant interest in the development of functional fruit products due to their potent antioxidant properties.

Furthermore, vitamin C content was negatively correlated with the number of segments (*r* = –0.44*), implying that fruits with fewer segments may accumulate higher amounts of vitamin C. This information may guide the selection and promotion of genotypes that meet consumer demands for nutrient-rich produce.

Collectively, these findings have important implications for breeding strategies, as well as for the tailored utilization of genotypes depending on the intended use. While small-fruited genotypes may be advantageous in terms of biofunctional properties for nutraceutical or industrial applications, larger, visually appealing fruits with favorable marketability may be prioritized for fresh consumption. Additionally, seed number and segment traits could inform rootstock selection strategies.

In conclusion, the significant correlations observed in this study contribute to the understanding of intraspecific variability and offer a scientific basis for multi-purpose genotype development. Such analyses are valuable tools in the efficient utilization of genetic resources and the advancement of targeted breeding programs.

Our findings are consistent with the results of a similar study conducted in Pakistan [31]. On the other hand, a similar study conducted in Sri Lanka reported a significant correlation between total phenolic content and total flavonoid content [1], suggesting that these two compounds may accumulate together through shared biosynthetic pathways. This finding provides biological support for the correlation observed in our study, reinforcing the validity of our results.

### Multiple regression analysis (MRA)

Based on the results presented in Table 6, the MRA reveals several significant associations between the dependent variables and their corresponding predictors among the studied sour orange genotypes. The analysis indicates that fruit width is the most influential predictor of fruit weight, with a *β* of 0.92 and a highly significant *p*-value (*p* ≤ 0.00). This strong and positive relationship implies that fruit width accounts for a significant portion of the variance in fruit weight, consistent with the notion that wider fruits tend to be heavier due to greater volume and tissue mass [53]. In contrast, mesocarp thickness shows a negative relationship with fruit weight (*β* = –0.17, *p* ≤ 0.00), suggesting that, when controlling for fruit width, an increase in mesocarp thickness may be associated with a decrease in overall fruit weight—possibly due to tissue distribution differences or internal density [28].

**Table 6:** The characteristics associated with pomological, morphological, and biochemical variables in the studied sour orange genotypes, as revealed using MRA and coefficients

Dependant character	Independent character	*r*	*r*^2^	*β*	*t* value	*p* value
Fruit weight	Fruit width	0.96^a^	0.93	**0.92**	21.56	0.00*
	Mesocarp thickness	0.98^b^	0.96	-0.17	-4.28	0.00
Number of seeds	Fruit length	0.44^a^	0.19	**0.44**	2.28	0.03*
Total phenolic content	Fruit width	0.61^a^	0.37	**-0.51**	-3.41	0.00*
	Total flavonoid content	0.75^b^	0.56	**0.44**	2.97	0.01*
Total antioxidant capacity	Fruit weight	0.53^a^	0.28	**0.53**	2.93	0.01*
Vitamin C content	Number of segments	0.44^a^	0.20	**-0.44**	-2.33	0.03*

*r*: Correlation coefficient; *r*^*2*^: Coefficient of determination; *β*: Standardized beta coefficients. *Multiple regression analysis correlations supported by the correlation matrix analysis

The number of seeds is significantly and positively affected by fruit length (*β* = 0.44, *p* ≤ 0.03), indicating that longer fruits tend to harbor more seeds [48]. This may reflect underlying developmental or genetic coordination between fruit elongation and seed formation, which can be of practical relevance for rootstock breeding, where seed yield and viability are key traits.

Biochemical characteristics also revealed informative associations. Total phenolic content was significantly and negatively associated with fruit width (*β* = –0.51, *p* ≤ 0.00). In larger fruits, the concentration of phenolic compounds per unit weight may decline due to a dilution effect. This phenomenon could be attributed to the expansion of fruit tissue due to increased cell division and/or water accumulation during fruit development [68]. A significant positive relationship was found between total phenolic content and total flavonoid content (*β* = 0.44, *p* ≤ 0.01), suggesting that these two compound groups tend to accumulate concurrently. This association may be due to their shared biosynthetic origin within the phenylpropanoid pathway. As flavonoids are a subclass of phenolic compounds, their parallel increase is biologically plausible. This relationship also implies that selecting for higher total phenolics may indirectly favor flavonoid-rich genotypes [42]. Such genotypes may be of particular interest for functional food production due to their enhanced antioxidant potential.

Furthermore, total antioxidant capacity showed a positive and significant association with fruit weight (*β* = 0.53, *p* ≤ 0.01), indicating that heavier fruits tend to possess higher antioxidant activity. This relationship underscores the potential of particular genotypes to combine favorable market traits (e.g., size, appearance) with nutritional value, which is particularly relevant for breeding programs targeting consumer preferences and functional food markets.

Finally, vitamin C content was significantly and negatively associated with the number of segments (*β* = –0.44, *p* ≤ 0.03), suggesting that fruits with fewer segments tend to accumulate more ascorbic acid. This relationship could have practical implications in selecting genotypes for enhanced nutritional quality, particularly in health-conscious markets or for use in juice processing industries. The bold values are supported by the correlation matrix analysis.

In summary, the multiple regression analysis revealed distinct predictive relationships among morphological, pomological, and biochemical traits. These results highlight the complex interplay between structural fruit traits and phytochemical attributes. The findings provide valuable insights for breeding strategies, enabling the identification of genotypes that balance yield, morphological quality, and biochemical richness. Future selection could be guided by these predictors, depending on the target use—whether for fresh consumption, rootstock production, or nutraceutical applications.

### Principal component analysis (PCA)

The results of the PCA demonstrate a clear and biologically meaningful structure within the dataset. The first five principal components collectively explained 82.44% of the total variance, with PC1, PC2, and PC4 being statistically significant at *p* < 0.01, and PC3 and PC5 at *p* < 0.05. Among these, PC1, PC2, and PC3 alone accounted for 68.44% of the total variance, indicating that a substantial proportion of the multidimensional variation in the data can be captured by just three components (Table 7).

**Table 7:** Eigenvalues of the principal component axes from the PCA of pomological, morphological, and biochemical variables in the studied sour orange genotypes

Eigenvectors	Component				
	1	2	3	4	5
Fruit weight	**0.37**^**a**^	0.18	0.13	0.18	0.04
Fruit length	**0.37**^**a**^	0.20	0.03	0.23	0.07
Fruit width	**0.37**^**a**^	0.19	0.01	0.21	0.08
Epicarp thickness	0.28	0.19	**-0.30**^**a**^	0.21	-0.20
Mesocarp thickness	−0.07	0.00	**−0.56**^**a**^	**0.35**^**a**^	**0.31**^**a**^
Number of segments	**0.32**^**a**^	−0.07	0.27	−0.24	0.17
Number of seeds	0.16	0.15	0.00	−0.13	**0.70**^**a**^
Leaf length	−0.18	**0.36**^**a**^	0.29	0.00	0.07
Leaf width	−0.08	**0.44**^**a**^	−0.02	−0.21	0.01
Leaf shape index	−0.15	−0.15	**0.48**^**a**^	**0.31**^**a**^	0.01
Leaf area	−0.14	**0.42**^**a**^	0.11	−0.12	0.04
Petiole length	−0.18	**0.39**^**a**^	0.12	0.07	−0.12
Petiole width	−0.10	**0.38**^**a**^	−0.26	−0.17	−0.24
Total phenolic content	**−0.34**^**a**^	0.01	0.03	0.13	0.20
Total antioxidant capacity	0.21	0.07	0.21	**0.30**^**a**^	**−0.37**^**a**^
Total flavonoid content	−0.23	0.10	0.17	**0.48**^**a**^	0.25
Vitamin C content	−0.22	0.04	−0.15	**0.31**^**a**^	−0.13
*Eigenvalue*	*5.35*	*4.55*	*1.74*	*1.22*	*1.16*
*Component degree of significance*	**	**	*	**	*
*Variance (%)*	*31.48*	*26.75*	*10.21*	*7.17*	*6.83*
*∑ variance (%)*	*31.48*	*58.23*	*68.44*	*75.61*	*82.44*

^**a**^Bold values indicate the characteristics that most influence each PC (Eigenvalues are significant ≥ 0.30). Component degree of significance: *p < 0.05, **p < 0.01

PC1, explaining 31.48% of the total variance, is predominantly associated with fruit morphological traits. The highest positive loadings were observed for fruit weight (0.37), fruit width (0.37), and fruit length (0.37), followed by number of segments (0.32). These traits likely represent a size-related dimension of fruit development. Interestingly, total phenolic content displayed a moderate negative loading (−0.34), suggesting an inverse relationship between fruit size and phenolic accumulation. This negative association may reflect a trade-off mechanism in which resources allocated to fruit enlargement limit the investment in phenolic biosynthesis. Such a trade-off aligns with the growth-defense hypothesis in plant biology, which posits that under resource constraints, allocation to growth and structural development may occur at the expense of secondary metabolite production.

PC2, which accounts for 26.75% of the variance, is primarily defined by vegetative morphological variables. Leaf width (0.44), leaf area (0.42), petiole length (0.39), petiole width (0.38), and leaf length (0.36) showed strong positive loadings, indicating that this component captures variability in leaf size and structure. The consistency and magnitude of these loadings suggest a coordinated expression of leaf growth traits, which are likely to be influenced by both genetic background and environmental conditions such as light, water availability, and nutrient status. These traits are functionally crucial for photosynthetic capacity and transpiration efficiency, and their co-variation within a single component reflects their interdependence in plant development and adaptation.

PC3 contributed 10.21% to the total variance and reflects a contrast between leaf shape and fruit tissue characteristics. The leaf shape index had a strong positive loading (0.48), while epicarp thickness (−0.30) and mesocarp thickness (-0.56) exhibited moderate to strong negative loadings. This pattern suggests that as leaf shape becomes more elongated or specialized, there may be a reduction in fruit pericarp thickness. The inverse relationship between these traits could point to underlying developmental or genetic trade-offs, where structural investment in leaf morphology may influence resource allocation patterns that affect fruit anatomy. Furthermore, the substantial negative loading of mesocarp thickness may indicate that this trait is under distinct regulatory control or may be influenced by different selective pressures compared to the vegetative traits captured in PC2.

The clear separation of traits among the first three components enhances the biological interpretability of the PCA results. PC1 highlights fruit size and phenolic balance, PC2 captures vegetative growth dimensions, and PC3 represents a divergence between leaf form and fruit tissue structure. The significant contribution of these components to total variance, along with their distinct loading patterns, provides a strong framework for genotype differentiation and trait-based selection. These findings underscore the utility of PCA in revealing complex phenotypic relationships and offer valuable insights into the potential physiological and developmental trade-offs inherent in plant systems.

A study conducted in France on sour orange reported that the first three principal components accounted for 68% of the total variation [20]. Similarly, a related study from Pakistan indicated that the first three components explained 92.51% of the total variation [31]. Our findings are in agreement with those reported by Ferrer et al. [20].

**Fig. 4:**
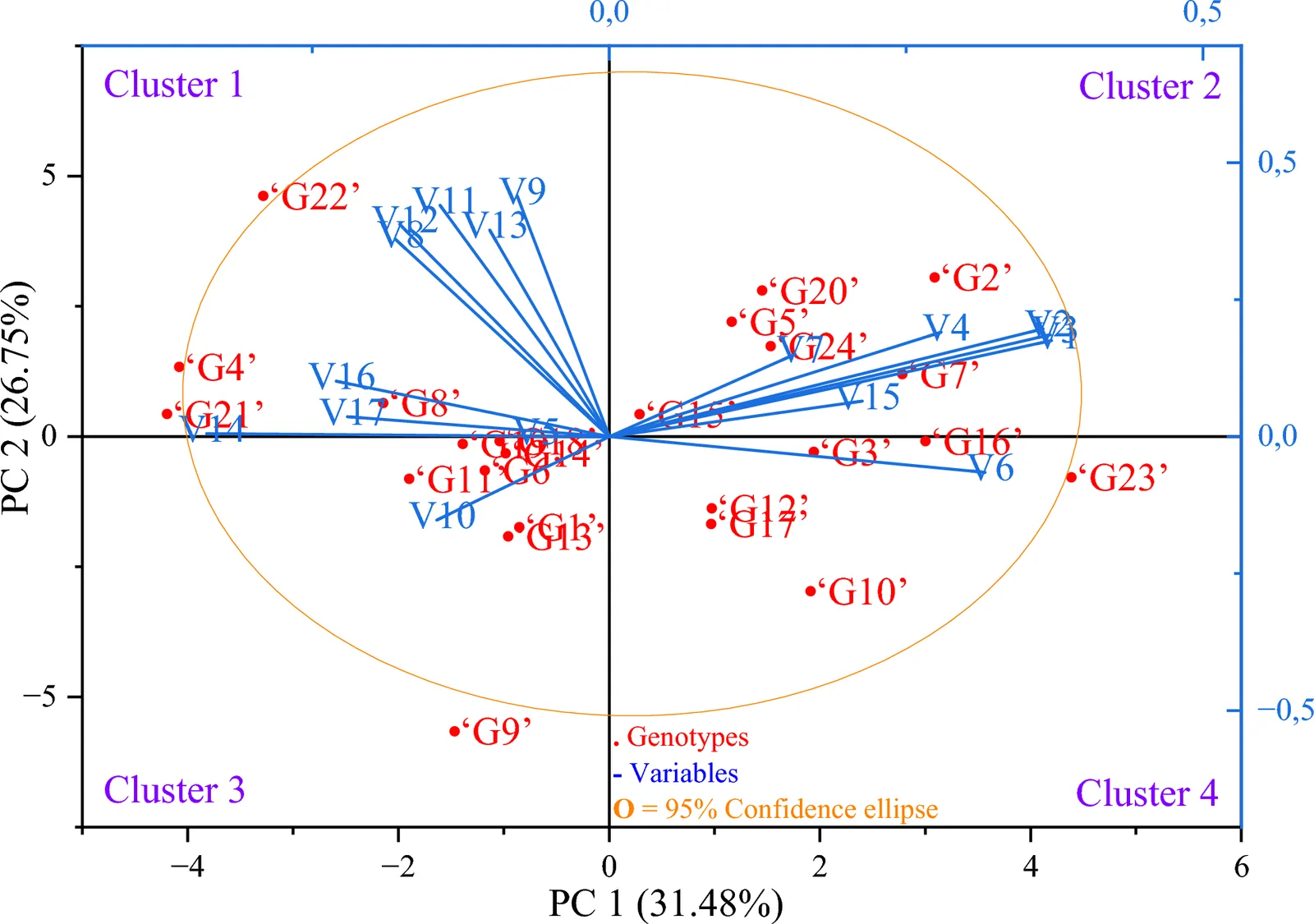
Biplot for the studied sour orange genotypes and variables based on PC1/PC2 of pomological, morphological, and biochemical traits. For abbreviations, please see Table 2

The biplot for the studied sour orange genotypes and variables, based on the first two principal components (PC1/PC2) of pomological, morphological, and biochemical traits, is presented in detail in Fig. 4. The first two principal components together account for 58.23% of the total variation, highlighting a substantial portion of the variability within the data set. The genotypes and variables are distributed across the four distinct regions of the biplot, which offers valuable insights into the relationships between the different traits and the genotypes.

Cluster 1, which includes genotypes ‘G4’, ‘G8’, ‘G21’, and ‘G22’, is characterized by a strong association with traits such as mesocarp thickness, leaf length, leaf width, leaf area, petiole length, petiole width, total phenolic content, total flavonoid content, and vitamin C content. This cluster suggests that these genotypes share similar morphological and biochemical characteristics, which could indicate their suitability for specific agricultural or breeding purposes where these traits are of importance, such as for improving fruit quality and nutritional value.

Cluster 2, consisting of genotypes ‘G2’, ‘G5’, ‘G7’, ‘G15’, ‘G20’, and ‘G24’, is associated with variables such as fruit weight, fruit length, fruit width, epicarp thickness, number of seeds, and total antioxidant capacity. These genotypes are particularly notable for their characteristics related to fruit size, structural integrity, and antioxidant potential, making them essential from the perspective of marketing criteria such as fruit size, texture, and health benefits. The clustering of these genotypes highlights their potential for selection in breeding programs to enhance fruit yield and quality. Additionally, these genotypes can be considered for use as rootstocks. Rootstock selection is critical for transferring traits related to yield, resistance, and overall plant health. Given their strong traits in fruit size and structural characteristics, these genotypes may serve as effective rootstocks to enhance the growth of scion plants, providing better resilience and vigor. The use of these genotypes as rootstocks could offer substantial benefits in terms of improving fruit production, as well as boosting plant resistance to environmental stresses, thus contributing to the overall health and productivity of sour orange cultivation.

Cluster 3, represented by genotypes ‘G1’, ‘G6’, ‘G9’, ‘G11’, ‘G13’, ‘G14’, ‘G18’, and ‘G19’, is notably associated with the leaf shape index. This cluster suggests that these genotypes may possess distinct leaf morphology, which could influence their overall plant growth and development. The leaf shape index is often a significant trait in plant breeding for pest resistance, canopy structure, and photosynthetic efficiency, making this group particularly relevant for studies focusing on plant architecture.

Cluster 4, consisting of genotypes ‘G3’, ‘G10’, ‘G12’, ‘G16’, ‘G17’, and ‘G23’, is associated with total flavonoid content. This cluster implies that these genotypes may have higher levels of flavonoids, which are compounds of interest due to their antioxidant properties and potential health benefits. The clustering of these genotypes around flavonoid content underscores their potential for further exploration in nutraceutical and functional food applications.

The biplot analysis revealed that the genotypes ‘G4’, ‘G9’, ‘G21’, ‘G22’, and ‘G23’ were positioned outside the 95% confidence ellipse, indicating significant divergence from the primary genotypic cluster. This deviation suggests that these genotypes possess unique trait combinations, distinguishing them from the broader population. Statistically, their outlier status implies they may represent distinct genetic backgrounds or harbor rare phenotypic expressions, making them particularly valuable for breeding programs seeking to expand genetic diversity or introduce novel traits. The observed divergence could stem from specialized adaptations, such as unique biochemical profiles, morphological features, or environmental resilience, which are not commonly observed in the main population. Given their distinctiveness, further agronomic and genetic characterization of these genotypes is warranted, as they may offer valuable traits for cultivar improvement or conservation strategies.

Overall, the biplot analysis effectively delineated genotypic relationships, highlighting clusters with shared morphological, pomological, and biochemical traits. This clustering provides critical insights for targeted breeding, enabling the enhancement of specific characteristics such as fruit quality, plant architecture, and nutritional content. The identification of outlier genotypes underscores their potential role in diversifying breeding material and advancing genetic research in sour orange populations.

The biplot distribution and the combined variance explained by PC1 and PC2 (57.2%) reported by Ferrer et al. [20] closely align with the findings of the present study in terms of both structural patterns and explained variation. Similar findings have been reported in a study conducted in Indonesia [35]. In both studies, genotypes and variables were distributed across four main regions, exhibiting a similar clustering tendency. This consistency indicates that the variation structure of the evaluated traits can be similarly represented across independent studies.

### Heat map analysis (HMA)

**Fig. 5:**
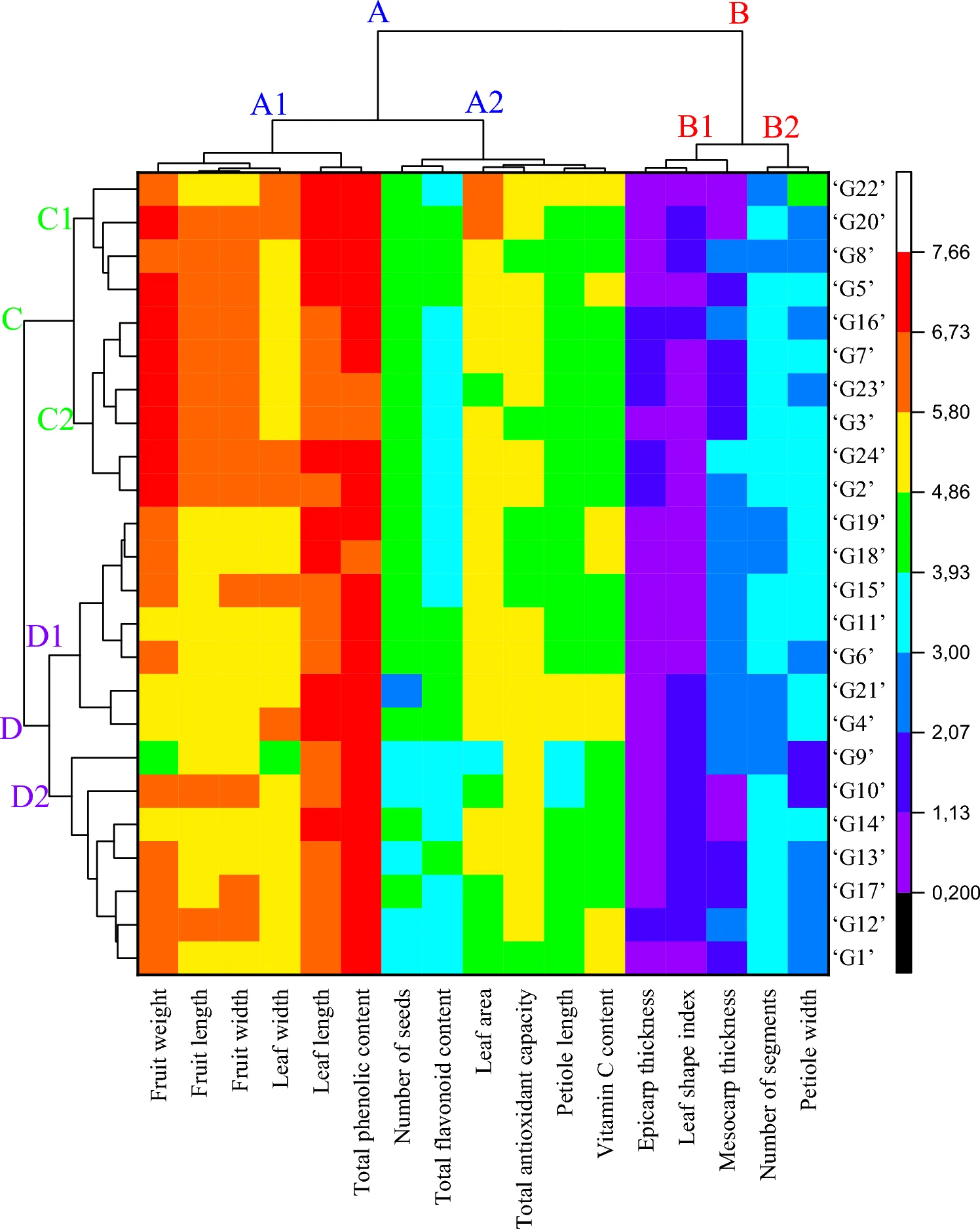
Visualization of clustering patterns of sour orange genotypes and variables based on pomological, morphological, and biochemical characterizations using a heat map

Figure 5 presents a heat map visualization illustrating the clustering patterns of sour orange genotypes and associated variables based on pomological, morphological, and biochemical characterizations. The hierarchical clustering grouped the variables into two primary clusters, A and B, which were subsequently divided into four subclusters: A1, A2, B1, and B2. Similarly, the genotypes were classified into two major groups, C and D, which were further subdivided into four subclusters: C1, C2, D1, and D2.

The A1 subcluster comprises variables related to fruit morphology and size, including fruit weight, fruit length, fruit width, leaf length, leaf width, and total phenolic content. The genotypes in the C1 subcluster (‘G22’, ‘G20’, ‘G8’, and ‘G5’) exhibit strong association with this group of variables, suggesting that these genotypes are characterized by larger fruit and leaf dimensions, as well as elevated phenolic content. This indicates a potential link between morphological development and the biosynthesis of phenolic compounds, possibly due to shared growth-regulating mechanisms.

The A2 subcluster includes the number of seeds, leaf area, petiole length, total antioxidant capacity, total flavonoid content, and vitamin C content. The genotypes in the C2 subcluster (‘G16’, ‘G7’, ‘G23’, ‘G3’, ‘G24’, and ‘G2’), associated with this group of variables, exhibit rich biochemical profiles, particularly characterized by high levels of antioxidant compounds and a correlation with the number of seeds. This suggests that these genotypes may possess higher metabolic activity and reproductive strategies, potentially contributing to increased resilience to environmental stress conditions.

In contrast, the B1 subcluster consists of epicarp thickness, mesocarp thickness, and leaf shape index. The D1 genotypes (‘G19’, ‘G18’, ‘G15’, ‘G11’, ‘G6’, ‘G21’, and ‘G4’) are closely aligned with this group, suggesting that these genotypes are defined by thicker fruit peel and more distinctive leaf morphology. These traits may contribute to mechanical protection, extended shelf-life, or environmental resilience, making these genotypes valuable for specific breeding objectives targeting fruit durability.

Lastly, the B2 subcluster includes a number of segments and petiole width. Genotypes in the D2 subcluster (‘G9’, ‘G10’, ‘G14’, ‘G13’, ‘G17’, ‘G12’, and ‘G1’) are most closely related to these variables. Although these traits may appear less prominent in terms of commercial traits, they could play a role in internal fruit structure and nutrient transport. They thus should not be overlooked in comprehensive evaluations.

In conclusion, the heat map analysis successfully identifies meaningful groupings among both genotypes and traits, revealing clear associations that reflect underlying biological and physiological relationships. These results not only contribute to understanding the diversity among sour orange genotypes but also provide a valuable basis for targeted breeding, conservation strategies, and genotype selection for specific pomological, morphological, or biochemical traits.

### Population analysis

**Fig. 6:**
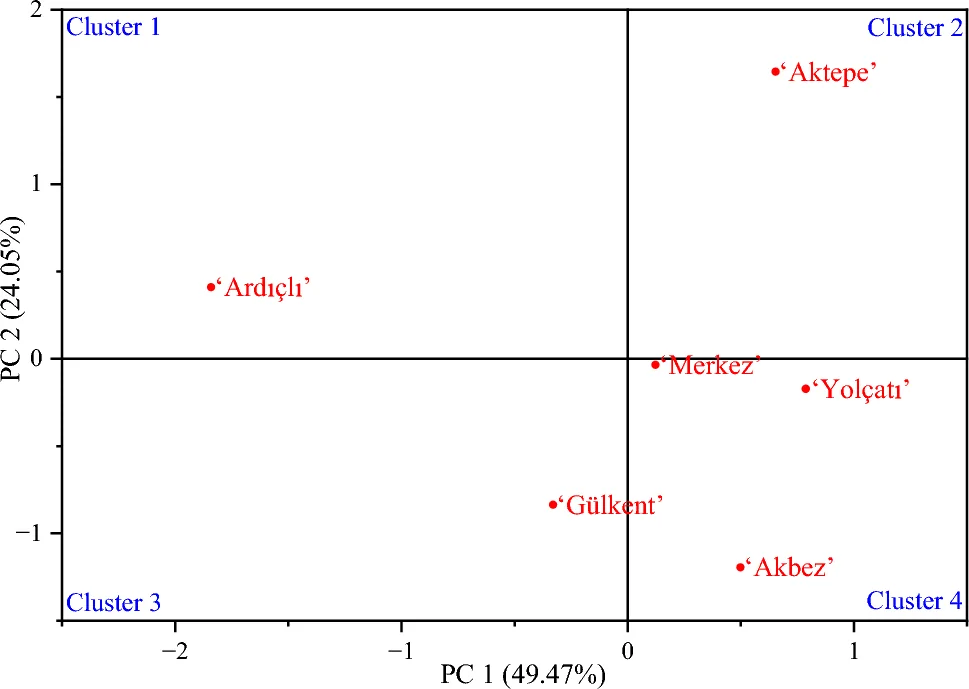
Scatter plot for the studied areas of sour genotypes based on pomological, morphological, and biochemical characterizations

In the context of population analysis, PC1 and PC2 accounted for 73.52% of the total variance. The population scatter plot revealed that the six populations were grouped into four distinct clusters. The first cluster contained ‘Ardıçlı’, the second cluster encompassed ‘Aktepe’, the third cluster included ‘Gülkent’, and the fourth cluster grouped ‘Merkez’, ‘Yolçatı’, and ‘Akbez’ (Fig. 6). Similar patterns of population structure have been previously documented in citrus germplasm using morphological, biochemical, and molecular markers. For instance, Curk et al. [12] reported strong genetic stratification among sour orange and related citrus taxa, reflecting both their complex domestication history and environmental adaptation. Likewise, Rohini et al. [54] emphasized that geographically proximate genotypes often clustered together, while populations with unique ecological pressures exhibited distinct genetic signatures. Our findings agree with these reports, as the close grouping of ‘Merkez’, ‘Yolçatı’, and ‘Akbez’ likely reflects shared genetic backgrounds due to geographic and historical connections, whereas the distinct placement of ‘Ardıçlı’ and ‘Aktepe’ suggests localized adaptation and possible genetic divergence. Population genetics, a well-established field in biology, traces its origins to the examination of hereditary trait diversity within populations and how such traits influence an organism’s adaptation to its environment [26].

### ISSR Analysis

In this study, 8 different ISSR primers were utilized to assess the genetic diversity of sour orange genotypes (Table 8). The total number of bands ranged from 5 [VHV(GTG)_7_ and (AG)_8_ T] to 13 [(CAC)_3_GC], resulting in a total of 139 scorable bands. Of these, 128 bands were identified as polymorphic, with the number of polymorphic bands varying between 5 [VHV(GTG)_7_ and (AG)_8_ T] and 11 [(CAC)_3_GC]. The average band length ranged from 190 to 1050 bp, with an average of 8.75 bands and 7.5 polymorphic bands per primer. The overall polymorphism rate was calculated to be 75.72%.

**Table 8:** ISSR primers were used to study sour orange genotypes

Primer	5’– > 3’ SEQUENCE	AT (°C)	BL (bp)	TBN	PBN	PR (%)
VHV(GTG)_7_	VHVGTGGTGGTGGTGGTGGTGGTG	50	300–750	5	5	100
(GT)6GG	GTGTGTGTGTGTGG	50	330–900	7	6	85.71
DBDA(CA)_7_	DBDACACACACACACACA	50	350–1050	11	10	90.91
(AG)_8_ T	AGAGAGAGAGAGAGAGT	50	450–1000	5	5	100
(CAC)_3_GC	CACCACCACGC	50	350–900	13	11	84.62
BDB(CA)_7_C	BDBCACACACACACACAC	50	200–950	8	8	100
(CAA)_6_	CAACAACAACAACAACAACAA	50	250–1050	11	6	54.55
(AGC)_6_G	AGCAGCAGCAGCAGCAGCG	50	190–1050	10	9	90
*Mean*	* –*	*50*	*–*	*8.75*	*7.5*	*75.72*
*Range*	*–*	*50*	*190–1050*	*5–13*	*5–11*	*54.55–100*
*Total*	* − *	*–*	*–*	*139*	*128*	*−*

*AT*: Annealing temperature; *BL*: Band length; *TBN*: Total band number; *PBN*: Polymorphic band number; *PR*: *(%)* Polymorphism rate

In a study conducted in Türkiye on lemon clones, RAPD (Random Amplified Polymorphic DNA) and ISSR markers were utilized to assess genetic diversity [61]. In this study, the number of bands obtained with RAPD primers ranged from 3 to 11, resulting in a total of 143 bands. The number of polymorphic bands ranged between 0 and 2, with only 8 polymorphic bands identified in total. The reported polymorphism rate varied from 0.0% to 25.0%, with an average of 5.6%. For the ISSR analysis, the number of bands ranged from 3 to 10, generating a total of 68 bands. Among these, 0 to 1 bands were found to be polymorphic, yielding a total of only 3 polymorphic bands. The polymorphism rate ranged from 0.0% to 20.0%, with an average of 4.4%. Yang et al. [64] conducted a study in China on 138 individual *Citrus hongheensis* trees and scored a total of 245 ISSR bands, corresponding to an average of 18.85 bands per primer. Among these, 233 loci were found to be polymorphic at the species level. The percentage of polymorphic loci (PPL) across all seven populations was reported to be 95.10%, indicating the high discriminatory power of ISSR markers in evaluating genetic diversity within citrus species. In a similar study conducted in Italy on cultivars of sour orange, ISSR markers were employed, and the total number of bands ranged from 3 to 12, yielding 53 bands in total. The number of polymorphic bands ranged between 1 and 6, with a total of 24 polymorphic bands identified. Additionally, the band sizes were reported to vary between 150 and 1500 bp [41]. In another study conducted in India on 38 genotypes of *Citrus jambhiri*, 40 SSR (Simple Sequence Repeats) primers were initially screened, and 17 SSR primers were selected for analysis based on reproducibility and banding patterns. A total of 60 bands were generated, all of which were determined to be polymorphic (100%). The total number of alleles ranged from 1 to 5, with an average of 3.52 alleles per locus reported [54].

**Fig. 7:**
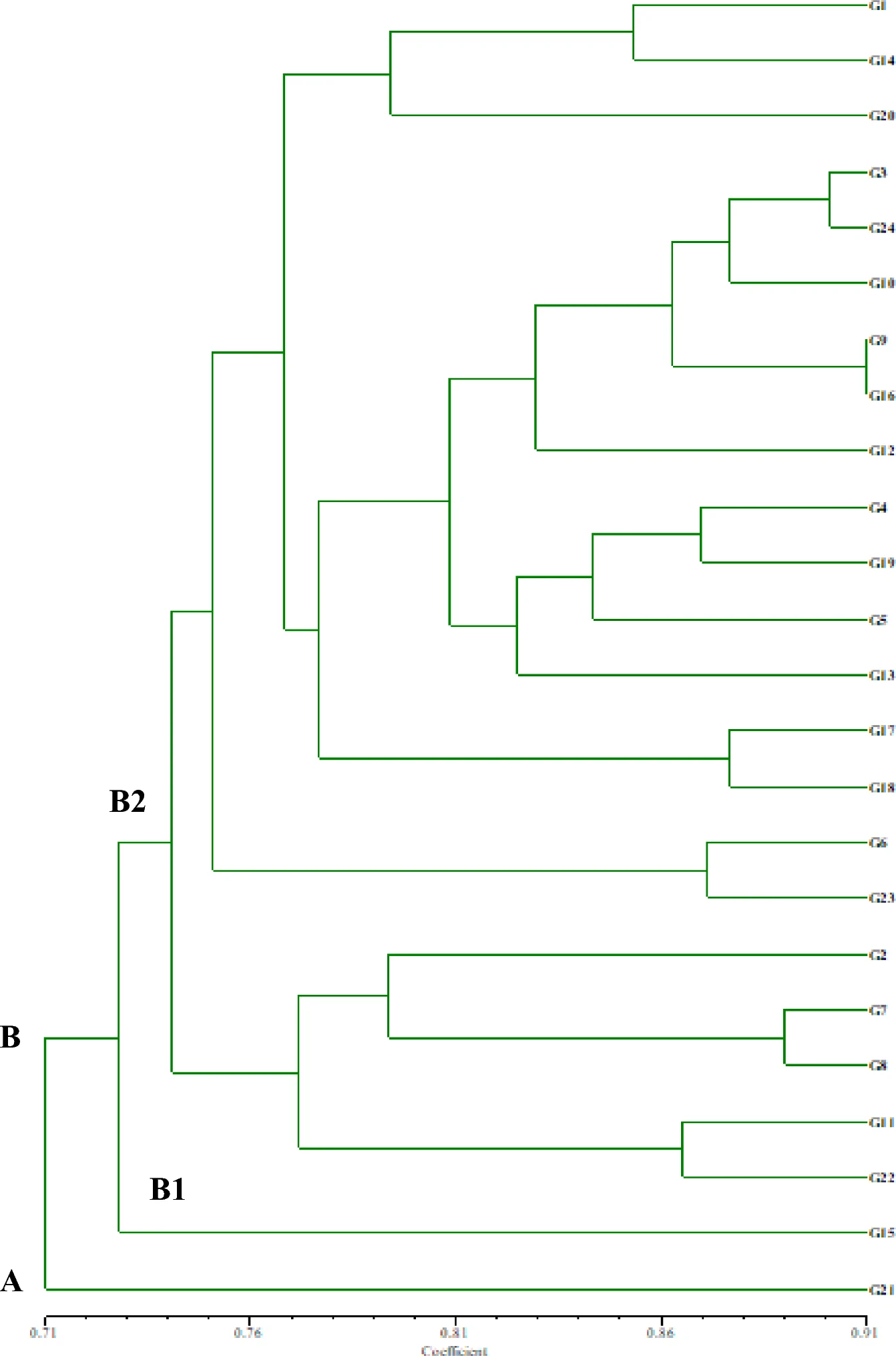
UPGMA dendrogram of sour orange genotypes based on ISSR data

In this study, the molecular diversity of sour orange genotypes was evaluated through a dendrogram constructed using the UPGMA method (Fig. 7). The similarity index among the 24 genotypes varied between 0.71 and 0.91. The dendrogram grouped the genotypes into two primary clusters. Only Group B was further divided into two subclusters. Group A consisted solely of the genotype ‘G21’, while Subcluster B1 contained only ‘G15’. The remaining genotypes were included in Subcluster B2. Among all genotypes, ‘G9’ and ‘G16’ exhibited the highest genetic similarity, with a similarity index of 0.91. Despite belonging to the same species, the genotypes showed both genetic relatedness and distinct variation. This genetic variability is likely due to natural gene exchange resulting from cross-pollination in the environment, as well as the use of ISSR markers, which target random regions across the genome [67]. Lombardo et al. [41] reported that, in a study conducted in Italy using ISSR markers, the similarity index among different sour orange cultivars ranged from 0.77 to 1.00, indicating a relatively high level of genetic similarity. Similarly, Ghahremani et al. [24] found that this index varied between 0.50 and 1.00 in their study conducted in Iran. The findings of our research fall within these ranges, suggesting that the genetic diversity observed among sour orange genotypes may vary depending on factors such as geographic origin, local cultivation practices, and the extent of natural hybridization.

Notably, the presence of genotype ‘G21’ as a single-member cluster in Group A suggests that this accession harbors a distinct genetic background compared with the other sour orange genotypes. Such solitary clustering patterns have been previously reported in citrus germplasm studies [12, 39] and are often attributed to unique allelic combinations, localized adaptation, or partial genetic isolation. This distinct placement highlights the potential importance of ‘G21’ as a unique genetic resource for broadening the genetic base in future citrus breeding and rootstock development programs.

The present analysis revealed a polymorphism rate of 75.72%, demonstrating substantial molecular variability among the sour orange genotypes. Notably, the unique clustering of genotypes ‘G21 and ‘G15’ indicates potential localized adaptation or genetic divergence, which is consistent with their phenotypic distinctiveness observed in morphological and biochemical assessments. Such findings underscore the capacity of ISSR markers to detect meaningful patterns of genetic differentiation, even when applied as a complementary tool alongside phenotypic and biochemical evaluations.

It is acknowledged that molecular population genetic studies often report indices such as expected heterozygosity (He), observed heterozygosity (Ho), allele frequency, and fixation index (FST). However, the primary aim of this research was to provide an integrative evaluation by combining morphological, pomological, biochemical, and molecular datasets, with ISSR serving a supportive rather than exhaustive role. Consequently, these indices were not computed. Nevertheless, the relatively high polymorphism rate (75.72%) and the dendrogram-based clustering patterns provide robust evidence of genetic variability among the genotypes. Although the dominant nature of ISSR markers—particularly their inability to distinguish heterozygous loci and the potential risk of homoplasy—constitutes a methodological limitation, ISSRs were selected in this study due to their high reproducibility and well-documented discriminatory power in *Citrus* species. The results obtained here are consistent with previous ISSR-based investigations in citrus germplasm [24, 41], underscoring the suitability of ISSRs for preliminary assessments of genetic diversity. Future studies should incorporate codominant markers such as SSRs and SNPs, as well as next-generation sequencing approaches, to enable higher-resolution molecular characterization and to provide more comprehensive insights into population genetic structure and evolutionary processes.

### AMOVA and population differentiation

The AMOVA results revealed that the majority of molecular variance was distributed within populations (87.12%), whereas only 12.88% of the total variance occurred among populations (Table 9). This indicates that genetic diversity in the wild sour orange genotypes of Hatay is predominantly structured at the individual level rather than being geographically partitioned.

**Table 9:** AMOVA based on the ISSR binary data matrix of sour orange genotypes

Source of variation	df	SS	MS	Variance component	Percentage of variance (%)
Among populations	5	41.63	8.33	2.11	12.88
Within populations	18	257.92	14.33	14.27	87.12
Total	23	299.55	−	16.38	100.00

ΦPT = 0.129; *p* = 0.001 (999 permutations). *Df*: Degrees of Freedom; *SS*: Sum of Squares; *MS*: Mean Squares

The ΦPT value calculated from ISSR markers was 0.129 (*p* = 0.001), demonstrating a low-to-moderate but statistically significant degree of genetic differentiation among the six sampling regions. These results suggest that although geographical separation exists, gene flow among natural sour orange populations has not been completely restricted. However, it should be noted that the relatively small number of genotypes per population (n = 4) may limit the statistical power of the AMOVA and influence the precision of the ΦPT estimate. In particular, small sample sizes can lead to increased sampling error and reduced ability to accurately partition genetic variance among and within populations. Therefore, the estimated level of genetic differentiation should be interpreted with caution.

## Conclusions

This study revealed substantial morphological, pomological, biochemical, and molecular diversity among wild sour orange genotypes collected from the Hassa district of Hatay, Türkiye. The pronounced phenotypic variability, together with the high ISSR polymorphism rate (75.72%), highlights the richness of this micro-region as a valuable reservoir of genetic resources with significant potential for rootstock breeding, fruit quality improvement, and nutraceutical applications. The predominance of within-population genetic variation (87.12%) further emphasizes that genetic diversity is largely maintained at the individual level, providing important implications for conservation and selection strategies. While the study was conducted within a single district and utilized dominant ISSR markers, these constraints do not diminish the robustness of the integrative dataset, which consistently revealed clear and biologically meaningful patterns of diversity. Similarly, although genotype × year interactions and detailed environmental variables were not explicitly modeled, the use of two-year averaged data provides a stable and representative assessment of the evaluated traits. Overall, the findings provide a reliable and comprehensive baseline for future sour orange research. Expanding the scope through broader geographic sampling, codominant molecular markers (e.g., SSRs and SNPs), and multi-year environmental analyses will further refine the understanding of genetic structure and adaptive variation in this species.

## Supplementary Information

Supplementary Material 1.

## Data Availability

The datasets generated and/or analysed during the current study are available from the corresponding author upon reasonable request

## Declarations

### Statement specifying permissions

For this study, we acquired permission to study sour orange issued by the Agricultural and Forestry Ministry of the Republic of Türkiye.

### Statement on experimental research and field studies on plants

The either cultivated or wild-growing plants sampled comply with relevant institutional, national, and international guidelines and domestic legislation of Türkiye.
